# Ureteritis Cystica: An Unusual Presentation in an Otherwise Healthy Female

**DOI:** 10.7759/cureus.2490

**Published:** 2018-04-17

**Authors:** Jai D Parekh, John Iguidbashian, Venkata Andukuri

**Affiliations:** 1 Internal Medicine, Creighton University Medical Center, Omaha, USA; 2 Creighton University Medical Center, Omaha, USA

**Keywords:** ureter, hydronephrosis, ureteritis cystica, nephrolithiasis, urinary tract infections (uti), acute kidney injury, ureter stenting

## Abstract

A 23-year-old, previously healthy female presented with lower abdominal pain and mildly elevated creatinine one month following a right ureter stent for non-specific ureteral thickening causing obstruction. On admission, computed tomography (CT) revealed moderate hydronephrosis of the left kidney that would require stent placement as well. During stent placement, it was noted that the gross appearance of the ureters resembled ureteritis cystica. Biopsies were taken and showed signs of chronic inflammatory changes consistent with this diagnosis. Interestingly, this patient had no obvious medical history suggesting a cause for this process. She had been otherwise healthy with no recurrent episodes of urinary tract infection, nephrolithiasis, or sexually transmitted infection. The patient was discharged symptom-free following stent placement and will follow with urology for future stent replacements and clinical monitoring.

## Introduction

Ureteritis cystica (UC) is a rare, benign condition of the ureters consisting of multiple, small submucosal cysts. It usually occurs in middle-aged to elderly females following chronic urolithiasis or recurring urinary tract infections. It has been hypothesized that the cause is the chronic inflammatory response secondary to a recurrent irritation of the mucosal lining of the ureters. Ureteritis cystica is very rare and, thus, is typically diagnosed incidentally while looking for other pathology. It generally requires no intervention in the absence of infection or obstruction. We present an unusual bilateral case of UC in a 23-year-old healthy woman with no history of the predisposing risk factors mentioned above.

## Case presentation

**Figure 1:**
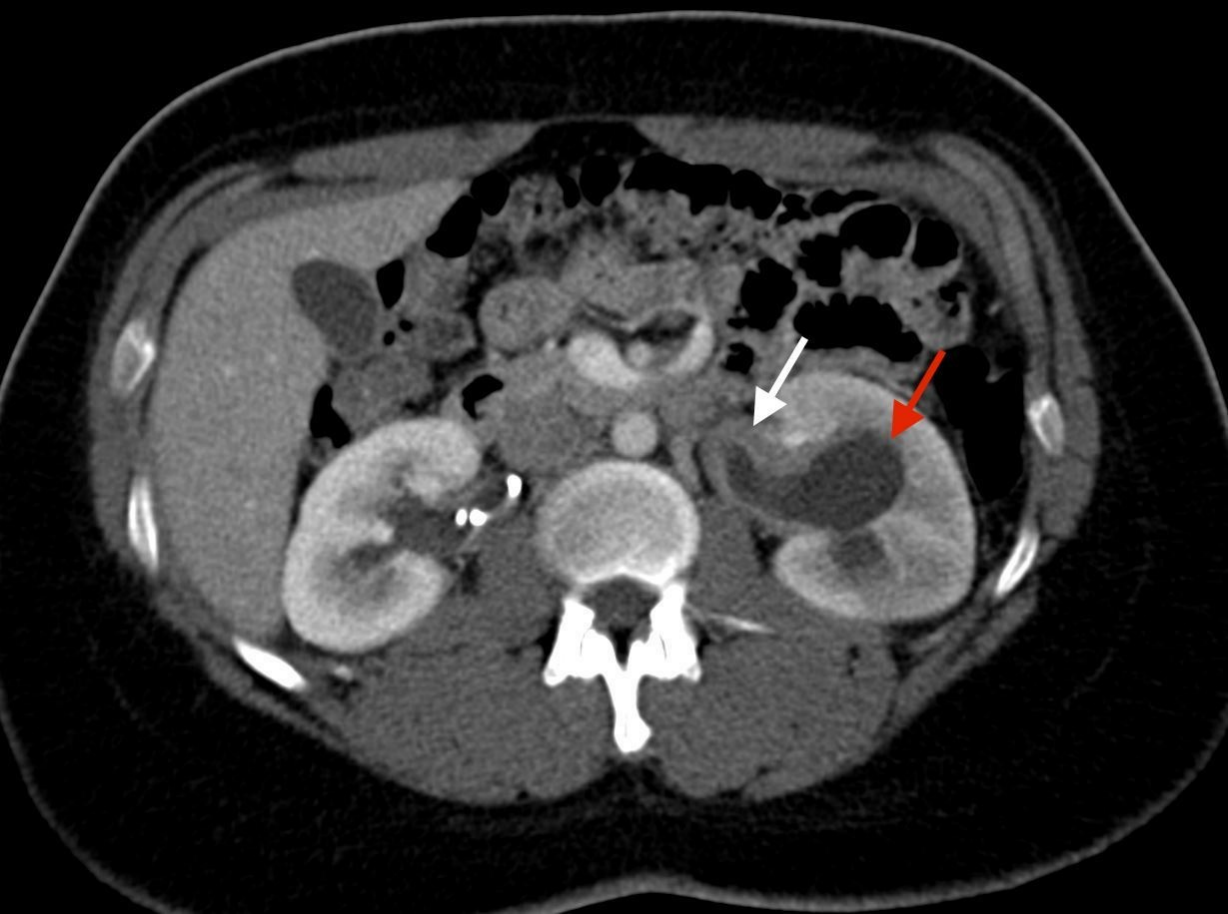
Axial CT image showing thickened left ureter (white arrow) and moderate hydronephrosis of the left kidney (red arrow) CT: computed tomography

**Figure 2:**
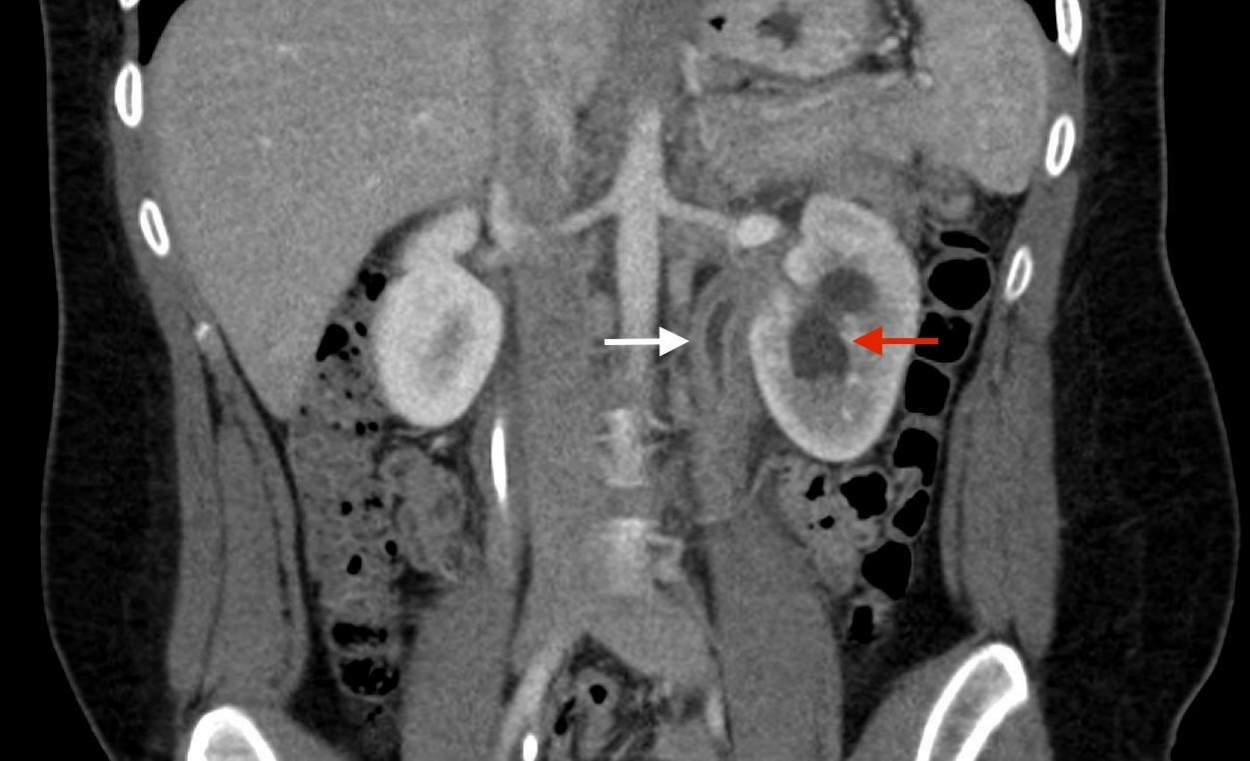
Coronal CT image showing thickened left ureter (white arrow) and moderate hydronephrosis of the left kidney (red arrow) CT: computed tomography

A 23-year-old woman presented with left lower quadrant abdominal pain and mildly elevated creatinine of 1.21 mg/dl (baseline 0.8-1 mg/dl) that was identified on routine blood draw at a one-month follow-up urology appointment. She had no urinary symptoms at that time and the review of systems was otherwise unremarkable. The physical examination was unremarkable without any focal abnormalities. Urinalysis and pregnancy testing were negative. Computed tomography abdomen revealed hydronephrosis of the left kidney as well as bilateral ureteral thickening (Figures 1-2). One month prior, she had a right ureter stent placed after she presented with acute kidney injury, and the CT abdomen at that time showed evidence of right hydronephrosis and bilateral proximal ureteral thickening. In addition, biopsies of ureter specimens found normal urothelium with signs of chronic inflammation during that initial hospitalization. Further workup was negative, including chest x-ray, sexually transmitted infection testing, complement activity levels, and other autoimmune markers. During this admission, a stent was placed in the left ureter and the right ureter stent was replaced. Repeat biopsies of both the right and left ureters showed findings of benign epithelial growth and chronic inflammation that were previously noted the month prior. Per the operative report, the region of the ureter thickening had the gross appearance of ureteritis cystica. However, upon further questioning, the patient had a history of only one urinary tract infection, no nephrolithiasis, and no other risk factors for this presentation. The patient was discharged home symptom-free and proceeded with close urological follow-up.

## Discussion

Morgagni first reported UC in 1761 [1]. It is a proliferative condition characterized by multiple cysts and filling defects in the urothelium. It has classically been documented in older female patients with a history of recurrent urinary tract infections or kidney stones and can be challenging to diagnose [2]. Most of the literature points toward unilateral ureteral involvement, with rare cases of bilateral findings, as described in our case.

UC may be associated with chronic urothelial irritation. Many studies have shown the causes of UC to be nephrolithiasis and urinary tract infections. Other etiological factors that have been postulated include schistosomiasis, vitamin A excess, and increased immunoglobulin A [2]. In one study, UC was found in a patient following formalin treatment for cyclophosphamide-induced hemorrhagic cystitis [3]. UC is usually asymptomatic; therefore, it is most frequently detected incidentally. The pathological features of UC include areas of glandular metaplasia secondary to chronic urothelial inflammation. The proliferation of epithelial bodies into the underlying mucosa leads to the formation of small cysts called von Brunn nests. The most common radiological appearance of UC is numerous, small, relatively uniform filling defects involving the ureters and, less commonly, the renal pelvis. The diagnosis is usually made during ureteroscopy or during radiography. Radiographically, a differential diagnosis of multiple transitional cell tumors, ureteral pseudodiverticula, non-opaque calculi, polyps, papillary tumors, tuberculosis, iatrogenic gas bubbles, gas-forming microorganisms, and submucosal hemorrhage can be considered with an appropriate clinical correlation [4]. Small filling defects and a bead-like appearance with regular surfaces in the ureter and renal pelvis are the typical findings demonstrated in intravenous or retrograde pyelogram. Magnetic resonance urography can provide high-resolution coronal images of the entire urinary tract without using contrast agents and ionizing radiation [5].

UC rarely presents with an ureteral obstruction or an acute kidney dysfunction according to the review of documented cases outlined by Padilla-Fernandez et al. [2]. This condition is more likely to present with symptoms of urinary tract infection, hematuria, or lithiasis before progression to obstruction occurs. There have been isolated cases reporting UC causing obstruction in the literature but this seems to be a rare occurrence [6-7]. Furthermore, Amos et al. reported a single case of bilateral UC that presented with unilateral ureteropelvic junction obstruction [8]. However, our patient developed bilateral ureter obstruction associated with an acute kidney injury, which has not been previously reported.

Various therapies can be applied in the treatment of UC. In 1946, Kopp obtained good results from the instillation of 2% silver nitrate into the ureters [9]. Petersen et al. recommended a conservative attitude using long-term antibiotics until normal radiography findings were obtained [10]. However, the potential side effects of antibiotics may warrant more cautious delivery of long-term therapy. Other treatment modalities include ureteral dilation or mechanical disruption of cysts and ureteral catheterization. However, more recently, a conservative approach of observation was recommended in the absence of infection or obstruction. Our patient presented with hydronephrosis during both instances and subsequently required stent placement.

## Conclusions

In conclusion, ureteral cystitis, although encountered on incidental diagnosis, should be considered during differential diagnosis, when there are small filling defects with a bead-like appearance along the urothelium of the renal pelvis, ureters, and bladder. This case may demonstrate an idiopathic origin of ureteritis cystica, although the bilateral and diffuse involvement, lack of previous urological history, and patient age are unusual and may warrant further workup towards other diagnoses. The etiology of ureteritis cystica is poorly understood and further investigations should aim to describe the typical presentation, diagnosis, and management guidelines for this process.
